# Supplementary material for: The Latest Prevalence, Isolation, and Molecular Characteristics of Feline Herpesvirus Type 1 in Yanji City, China
Source: Vet Sci. 2024 Sep 7;11(9):417. doi: 10.3390/vetsci11090417 (PMC11435738; doi:10.3390/vetsci11090417)
Supplement: Supplementary file 1 [file vetsci-11-00417-s001.zip › vetsci-3165952-supplementary.pdf]

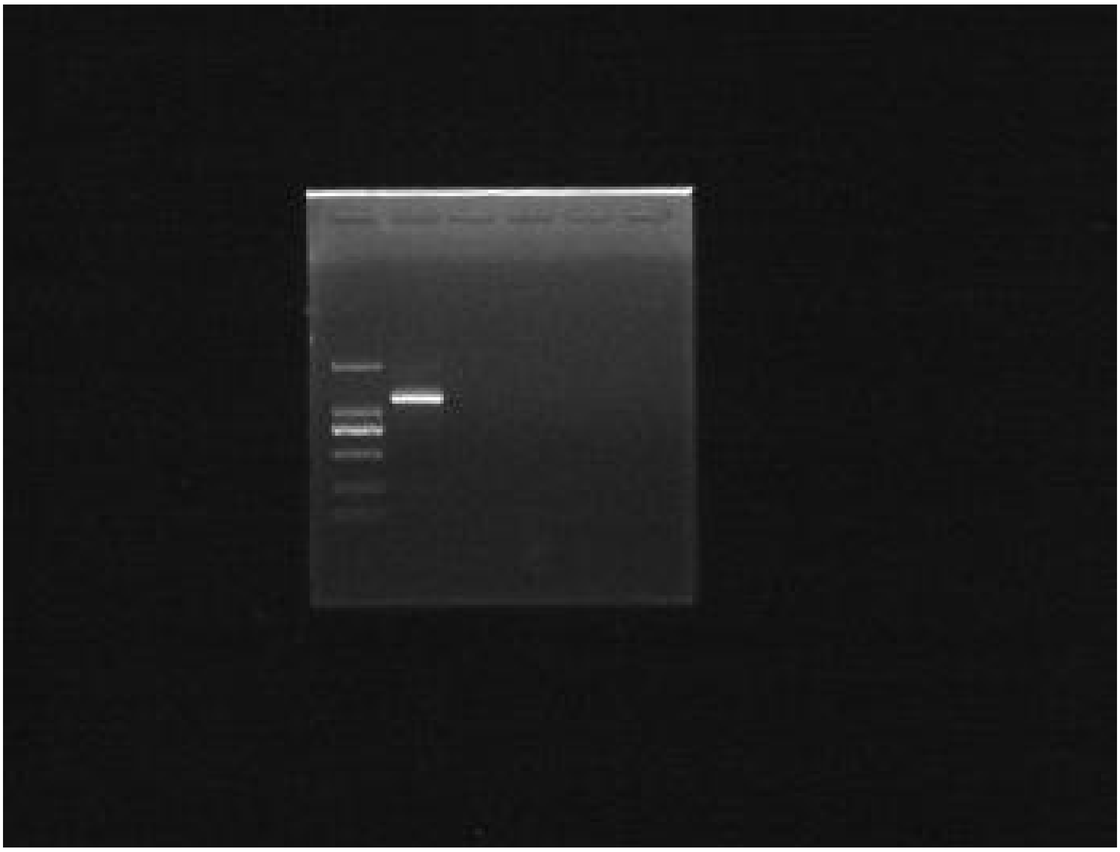

Figure S1. Identification results of the isolates, Lane M, DL 2000 Marker; Lanes 1, Amplified products of the isolates; Lanes 2-5: FPV, FCV, FCoV, and negative control.

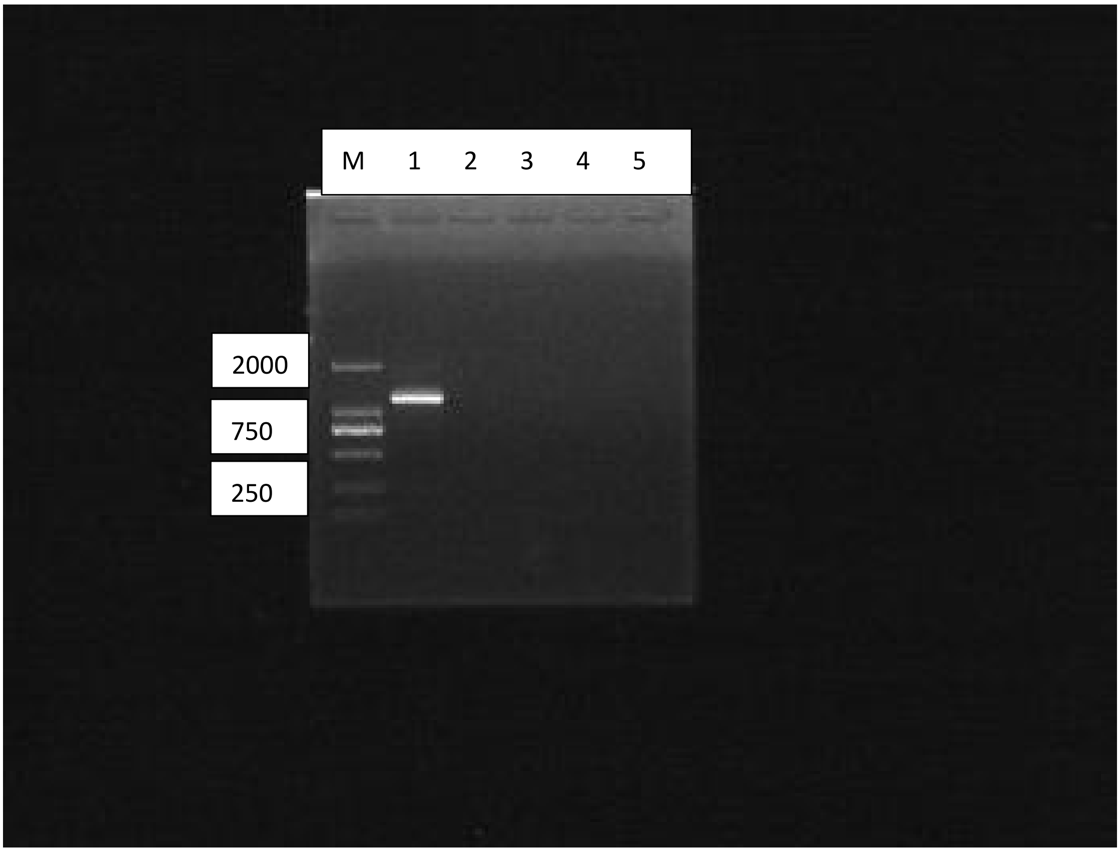

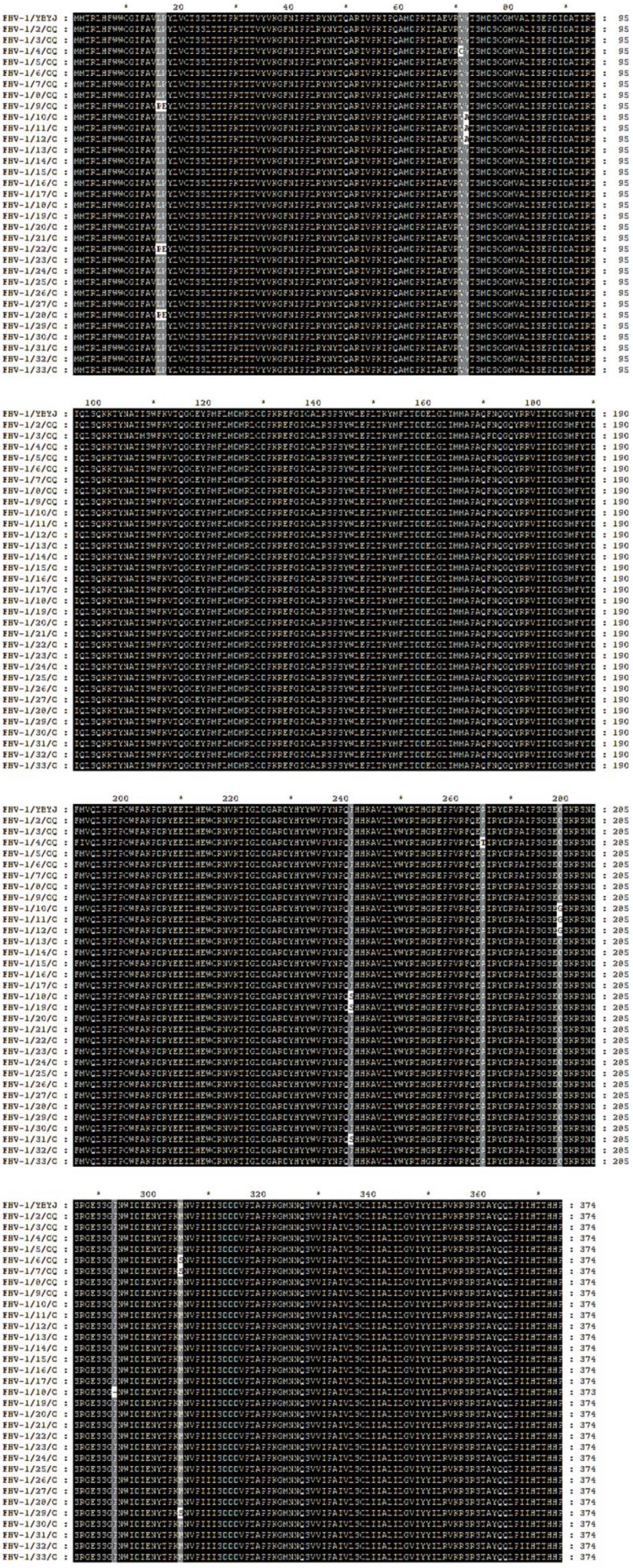

Figure S2. Sequence alignment between the 33 FHV-1 strains involved in the study

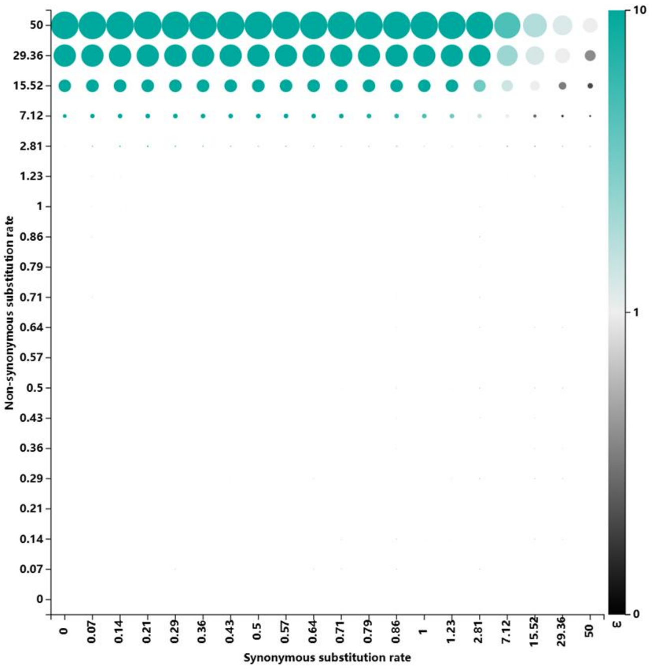

Figure S3. Analysis of amino acid selection pressure at position 241 of the gD gene.

Table S1. All FHV-1 negative samples were detected in this epidemiological survey.

Name	Breed	Age (month)	Clinical symptoms	Vaccine history
34	Orange cat	2	Sneeze	−
35	Pastoral cat	9	Dispirited	+
36	American Shorthair	3	Fever	○
37	British short-haired cat	7	Sneezeing	+
38	Pastoral cat	3	Conjunctivitis, sneezing	○
39	Chinese Li Hua	2	Cough	−
40	Maine	6	Conjunctivitis	−
41	British short-haired cat	8	Dispirited, sneezing	+
42	American Shorthair	7	Eye secretions, sneezing	+
43	Sphynx	2	Snot, sneezing	−
44	British short-haired cat	6	Sneezeing	−
45	American Shorthair	10	Dispirited, sneezing	+
46	British short-haired cat	7	Conjunctivitis	+
47	Maine	5	Sneezeing	−
48	Chinese Li Hua	5	Dispirited	−
49	British short-haired cat	12	Sneezeing	+
50	American Shorthair	4	Conjunctivitis, sneezing	−
51	British short-haired cat	3	Dispirited, fever	○
52	Garfield	7	Dispirited, sneezing	+
53	Pastoral cat	3	ocular secretions	○
54	Chinese Li Hua	2	conjunctivitis	−
55	Maine	6	ocular secretions	−

56	Pastoral cat	9	Fever, sneezing	+
57	American Shorthair	4	ocular secretions	−
58	Ragdoll	2	Dispirited, fever	−
59	Pastoral cat	3	Conjunctivitis	○
60	British short-haired cat	15	ocular secretions	+
61	Chinese Li Hua	5	Cough	−
62	British short-haired cat	8	Conjunctivitis, sneezing	+
63	Orange cat	5	Dispirited	−
64	American Shorthair	10	Sneezeing	+
65	British short-haired cat	7	ocular secretions	−
66	American Shorthair	8	Conjunctivitis	+
67	Sphynx	4	Conjunctivitis, sneezing	○
68	Orange cat	2	ocular secretions	−
69	Maine	6	Sneezeing	−
70	Ragdoll	2	Dispirited	−
71	Garfield	11	Conjunctivitis, sneezing	−
72	Siamese	12	Dispirited, sneezing	+
73	Pastoral cat	5	Sneezeing	−
74	American Shorthair	9	Conjunctivitis	−
75	British short-haired cat	6	Cough	−
76	Maine	3	Dispirited	○
77	Orange cat	2	Dispirited	−
78	Chinese Li Hua	2	Cough	−
79	American Shorthair	4	Sneezeing	○
80	British short-haired cat	8	Dispirited	+
81	Siamese	12	Cough, fever	+
82	Maine	6	Conjunctivitis, sneezing	−
83	Pastoral cat	3	Conjunctivitis	○
84	British short-haired cat	5	Dispirited, sneezing	−
85	American Shorthair	7	Dispirited	+
86	Chinese Li Hua	2	ocular secretions	−
87	American Shorthair	3	Cough	○
88	American Shorthair	6	Conjunctivitis	−
89	British short-haired cat	5	Sneezeing	−
90	Orange cat	8	Dispirited, sneezing	+
91	American Shorthair	3	ocular secretions	○
92	Pastoral cat	9	Dispirited	−
93	Maine	5	Conjunctivitis	−
